# Enorme tuméfaction mammaire bilatérale révélant une leucémie aigue (a propos d'un cas)

**DOI:** 10.11604/pamj.2016.23.41.7222

**Published:** 2016-02-12

**Authors:** Lamiae Boutkhil, Moulay Abdelilah Melhouf

**Affiliations:** 1 Service de Gynéco-Obstétrique 2, CHU Hassan II, Fès, Maroc

**Keywords:** Tuméfaction mammaire bilatérale, mastite, leucémie

## Image en medicine

C'est une patiente âgée de 27 ans sans antécédents pathologiques notables G2P2 qui consulte à j + 12 du post partum pour augmentation importante et rapide du volume des deux seins. L'examen clinique trouve une énorme tuméfaction mammaire bilatérale sans signe inflammatoire ni écoulement mammaire. La patiente a été mise sous anti œdémateux en attendant le rendez vous de l’échographie mammaire. Entre temps soit J + 35 elle a présenté une dyspnée stade IV sans douleur thoracique avec à l'examen: persistance de l'augmentation du volume mammaire, des adénopathies cervicales bilatérales, une adénopathie axillaire gauche 3cm, une adénopathie inguinale gauche et une hypertrophie gingival dans un contexte d'altération de l’état générale. Echographie mammaire: mastite bilatérales avec adénopathies axillaires bilatérales suspectes. Radiographie du thorax épanchement liquidien de moyenne abondance gauche Scanner thoraco-abdomino-pelvien: adénopathies axillaires bilatérales et médiastinales associée à une hyperplasie thymique pouvant être en rapport avec une origine lymphomateuse. Numération formule sanguine: Hémoglobine: 6,3g/dL plaquette: 42000/uL, globules blancs à 35 000/uL dont 40% de blastes lymphocyte: 0 plynucléaires neutrophiles: 2. Le diagnostic d'une leucémie aigue à été posé. Les leucémies aiguës sont des affections malignes du tissu hématopoïétique, caractérisées par la prolifération clonale de précurseurs hématopoïétiques. Une hypertrophie des organes hématopoïétiques: adénopathies, hépatomégalie, splénomégalie sont fréquentes mais aucun cas n'a été décrit d'hypertrophie de la glande mammaire. Nous rapportons un cas historique de leucémie aigue révélée par une énorme tuméfaction mammaire bilatérale survenue en post partum.

**Figure 1:**
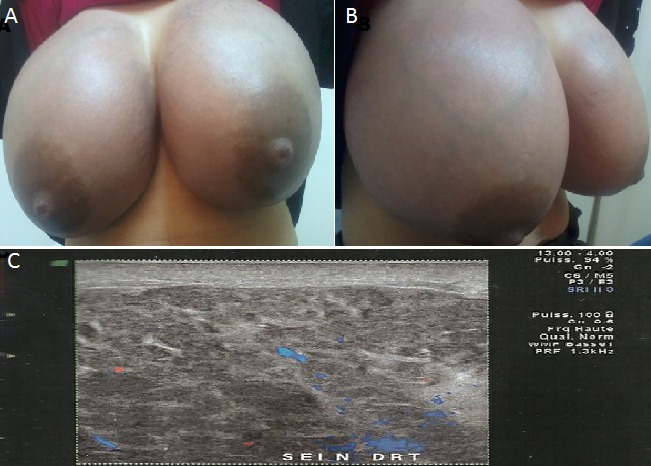
Tuméfaction mammaire bilatérales révélant une leucémie aigue. (A) seins tuméfiés vue de face; (B) seins tuméfiés vue de profil; (C) échographie mammaire aspect de mastite

